# Efficiency and Fidelity of Site-Directed Mutagenesis with Complementary Primer Pairs

**DOI:** 10.3390/cells15020138

**Published:** 2026-01-13

**Authors:** Paulina Varela-Castillo, Arezousadat Razavi, Negar Mousavi, Nicole Robinson, Xiang-Jiao Yang

**Affiliations:** 1 Rosalind and Morris Goodman Cancer Institute, McGill University, Montreal, QC H3A 1A3, Canada; arezousadat.razavi@mail.mcgill.ca (A.R.); 2 Department of Medicine, McGill University, Montreal, QC H3A 1A3, Canada; 3 Department of Biochemistry, McGill University, Montreal, QC H3G 1Y6, Canada; 4 Department of Medicine, McGill University Health Center, Montreal, QC H4A 0B1, Canada

**Keywords:** ClinVar variant, neurodevelopmental disorder, cancer, epigenetic regulator, BRPF1, BRD1, JADE2, KAT2B, p300, genome editing, Cas9

## Abstract

**Highlights:**

**What are the main findings?**

- This study improves the QuickChange site-specific mutagenesis method and makes it faster and more reliable by replacing Pfu with SuperFi II and Q5 DNA polymerases.
- Analysis of the failed plasmids uncovers frequent insertions of oligonucleotide repeats at the primer sites, thereby identifying a novel molecular mechanism by which partially overlapping primers with 3′-overhangs enhances mutagenesis efficiency to the ideal level of ~100%.

**What are the implications?**

- While still less efficient than P3a and P3b mutagenesis, the improved method is very reliable for engineering point mutations and small insertions or deletions.
- The frequent insertion of short repeats at the primer sites during site-directed mutagenesis with completely overlapping primers not only points to a new direction on how to improve this and related methods and enhance the mutagenesis efficiency further but also sheds light on how insertional mutations occur in cancer and other diseases.

**Abstract:**

Based on PCR with complementary primer pairs and Pfu DNA polymerase, QuickChange site-directed mutagenesis has been widely employed, but its efficiency varies from mutation to mutation. An alternative strategy relies on partially overlapping primer pairs with 3′-overhangs, and this strategy has led to the recent development of P3a and P3b site-directed mutagenesis, in which the use of SuperFi II and Q5 polymerases raises the mutagenesis efficiency to ~100%. It is unclear whether these two DNA polymerases also improve the QuickChange method. Herein, we have evaluated this possibility by engineering 46 mutations on seven expression plasmids, two of which possess extremely GC-rich sequences. As Pfu DNA polymerase is a slow enzyme, its replacement with SuperFi II and Q5 polymerases reduced PCR length. Moreover, the average efficiency for each of the seven plasmids ranged from 48% to 69%, thereby outperforming the original QuickChange method. However, this efficiency is still lower than that from the P3a and P3b methods, supporting the superiority of primer pairs with 3′-overhangs. Analysis of the incorrect plasmids from the improved QuickChange method revealed frequent insertions at primer sites. The insertions were derived from primers and varied from mutation to mutation, with certain sites much more prone to such insertions. In comparison, these insertions occurred at a much lower frequency with the P3a and P3b methods, suggesting that primer pairs with 3′-overhangs enhance mutagenesis efficiency by reducing the likelihood to introduce insertions at primer sites. Thus, this study improves the QuickChange mutagenesis method, supports the superiority of the P3a and P3b methods, and uncovers a novel molecular mechanism by which the efficiency of PCR-based mutagenesis with completely overlapping primer pairs is negatively affected.

## 1. Introduction

Site-directed mutagenesis is a basic molecular biology tool for engineering gene mutations and testing their functional impact in vitro. Among all methods that have been developed since the principle was first introduced almost five decades ago [1,2], the QuickChange™ site-directed mutagenesis method has emerged as the most widely used [3,4,5,6]. It relies on Pfu (the hyperthermophilic archaeon *Pyrococcus furiosus*) DNA polymerase-mediated PCR with a pair of complementary primers, followed by DpnI digestion to selectively degrade wild-type plasmid templates that possess methylated GATC sites for DpnI recognition [3,4,5,6,7]. As both primers of the complementary pair encode the desired mutation, the maximal theoretical mutagenesis efficiency is 100%. But in practice, the efficiency varies from mutation to mutation and from plasmid to plasmid, thereby requiring considerable efforts to screen for the correct mutants after mutagenesis reactions. In some cases, lots of troubleshooting efforts are needed. Understanding the reasons behind the varied efficiency should help optimize the method, but little is currently known in this regard. It is thus necessary to carry out additional research to identify the underlying mechanisms for standardizing the procedures for all plasmids and mutations. The ideal goal is to achieve consistent high efficiency, at or near 100%, for all mutations and plasmids, to save time and reduce costs.

A more innovative strategy is P3 (primer pairs with 3′-protruding ends) site-directed mutagenesis based on partially complementary primer pairs that possess 3′-overhangs [8,9,10]. This innovative primer-designing strategy relies on the hypothesis that unlike QuickChange primers, primer pairs with 3′-protruding ends allow the primers to anneal to newly synthesized DNA strands and utilize them as templates for subsequent PCR amplification [8,9,10]. However, this hypothesis has never been tested. Thus, we decided to investigate this hypothesis experimentally by utilizing the above two types of primers to engineer the same sets of mutations. As describe below, the results indicate that there is an alternative molecular mechanism by which P3 primers are much more successful than QuickChange primers.

We have recently optimized the P3 mutagenesis strategy and improved its efficiency to the ideal level at or close to 100% [11,12,13]. The resulting method has been referred to as P3a site-directed mutagenesis, which, in addition to point mutations, allows seamless and high-efficiency deletion, insertion and replacement of DNA fragments thanks to the unique “hand-shaking” feature of P3 primer pairs [13,14]. One major modification in the new method is the replacement of Pfu DNA polymerase with SuperFi II and Q5 DNA polymerases to leverage their higher fidelity and faster synthesis rates [13,14]. Moreover, we have found that GC-rich sequences impede P3a site-directed mutagenesis and likely all other known mutagenesis methods [11,12,13,15,16], at least in part due to formation of guanine (G)-quadruplexes [17,18,19,20]. As a result, we have developed P3b site-directed mutagenesis, to address the challenges posed by extremely GC-rich sequences [21].

An interesting but unaddressed question is whether the replacement of Pfu DNA polymerase with SuperFi II and Q5 DNA polymerases also improves the original QuickChange method, which relies on completely complementary primer pairs (rather than the partially complementary primer pairs with 3′-overhangs used in the P3a and P3b methods) and has been widely used in many laboratories (including our own) around the world. To investigate this issue and provide direct comparison to the P3 primer-designing strategy, we assessed the use of these two enzymes in combination with QuickChange primers. We evaluated the resulting new method, referred to as QuickChange version 2.0 (or QC2) mutagenesis, by engineering 46 mutations on seven different plasmids expressing six epigenetic regulators and a Cas9 (CRISPR-associated protein 9) genome-editing endonuclease. Notably, two such plasmids possess extremely GC-rich sequences, which are known to impede site-directed mutagenesis [8,9,12], perhaps because of formation of G-quadruplexes [17,18,19,20]. The average efficiency for these seven plasmids ranged from 48% to 69%, which was better than the original QuickChange method but still much lower than that from P3a or P3b mutagenesis [13,21], thereby supporting the superiority of the P3 primer-designing strategy. Moreover, another advantage of this strategy is the ability of such primers to introduce small or large deletion, insertion and replacement [13,21].

Importantly, analysis of the incorrect plasmids from the QC2 method revealed frequent insertions at the primer sites, with certain primer sites more prone to such insertions, perhaps due to unique DNA sequences at those sites. By comparison, such insertions rarely occurred with P3a and P3b mutagenesis [13,21], suggesting that primer pairs with 3′-overhangs lead to high-efficiency mutagenesis at least in part because of their low likelihood to introduce insertions at the primer sites. This novel mechanism is completely different from the accepted notion that primer pairs with 3′-overhangs enhance mutagenesis efficiency by allowing the use of newly synthesized strands as templates for subsequent PCR amplification [5,6,8,9,10]. Therefore, the current study not only improves efficiency of the QuickChange mutagenesis method but also uncovers a novel mechanism that negatively affects the efficiency of mutagenesis based on PCR with complementary primer pairs.

Because of our own research interests, we have only tested the QC2 method with six epigenetic regulators and the genome-editing enzyme Cas9, but because the plasmids are of different sizes (up to ~13 kb) and possess different GC-contents (with extremely high GC-rich regions, close to 95%, in two vectors), we anticipate that the method will be applicable to many other proteins and plasmids. This study complements the recent works on P3a and P3b mutagenesis [13,21] and helps standardize mutagenesis experiments to reduce the failure rate greatly and minimize the need for troubleshooting efforts. It is also anticipated that once developed in the future, enzymes equivalent to or better than SuperFi II and Q5 DNA polymerases will be suitable for replacing these two enzymes in the QC2, P3a and P3b methods. These new three methods offer an economical and fast alternative to generation of mutants via total chemical synthesis [22]. In addition to traditional protein and plasmid targets, these three methods will be valuable for drug development based on rapid advances in artificial intelligence (AI)-assisted protein design [23,24,25,26,27].

## 2. Materials and Methods

### 2.1. Plasmids

Mammalian expression vectors for HA-tagged BRPF1 (bromodomain- and PHD finger-containing factor 1), BRPF2 (also known as BRD1, bromodomain protein 1), BRPF3, JADE2 (Jade family PHD finger 2), KAT2B (lysine acetyltransferase 2B, formerly known as PCAF) and p300 (adenoviral E1A-associated cellular protein of 300 kDa) were described previously [13,28]. The Cas9 expression plasmid, eSpCas9(1.1)_No_FLAG_ATP1A1_G3 (a pX330-derived vector [29,30]), was purchased from Addgene (#86611, Addgene, Watertown, MA, USA). For heat denaturation, 20 μL of the KAT2B or Cas9 expression plasmid (0.1 μg/μL with 1 × NEB restriction digestion buffer #2, Cat. B7002S, New England Biolabs, Ipswich, MA, USA) was incubated at 105 °C for 5 min before rapidly cooling on ice.

### 2.2. Site-Directed Mutagenesis

P3 site-directed mutagenesis based on Pfu_Ultra DNA polymerase and P3a or P3b mutagenesis relying on SuperFi II and Q5 DNA polymerases were carried out as previously described [11,13,21]. PCR conditions for classical QuickChange mutagenesis were identical to those described for P3 mutagenesis [8], but completely complementary primers were used. For QC2 mutagenesis, PCR conditions were identical to those used for P3a and P3b mutagenesis [10,12], except that completely complementary primers were utilized. Notably, PCR conditions for QC2 mutagenesis with plasmids containing GC-rich sequences, such as thoses for KAT2B and Cas9, were similar to those used in the P3b method rather than the classical QuickChange mutagenesis method.

Mutagenic primers were designed with aid of the SnapGene software package (version 8.0) and synthesized at Integrated DNA Technologies (Coralville, IA, USA) as 25-nmol scale desalted DNA oligos, with their sequences listed in Table S1. All oligonucleotides were used without polyacrylamide gel or HPLC purification. Upon receipt from Integrated DNA Technologies, autoclaved Nanopure water was added to each tube to dissolve lyophilized oligonucleotides and prepare 100 μM (i.e., 100 pmol/μL) stocks for further dilution to 5 μM (i.e., 5 pmol/μL) working solutions before setting up mutagenesis reactions [11,13,21]. To simplify pipetting, the forward and reverse primers for each mutation were mixed in one tube to form a working solution, whichsaved time and avoided pipetting errors when there were multiple mutagenesis reactions to be carried out in one experiment.

### 2.3. Statistics

Considering the qualitative nature of typical mutagenesis experiments (e.g., just needing to obtain only one mutant plasmid per mutagenesis reaction), we focused on evaluating efficiency variations from mutation to mutation and from gene to gene. Means and standard deviations were computed via an online calculator: https://www.calculator.net/standard-deviation-calculator.html (accessed on 2 and 24 December 2025). Due to the qualitative nature, the success rates of the QC2 method were compared with that from the original QuickChange method via a one-proportion Z test: https://www.statology.org/one-proportion-z-test-calculator/ (accessed on 21 December 2025). Student’s T tests were performed to compare QC2 and P3a methods via Microsoft Excel (version 16.104) or an online tool: https://www.graphpad.com/quickcalcs/ttest1/ (accessed on 21 December 2025), with *p* values smaller than 0.05 considered statistically significant.

## 3. Results

### 3.1. Developing QC2 Mutagenesis for Engineering Point Mutants of BRPF1

**Figure 1:**
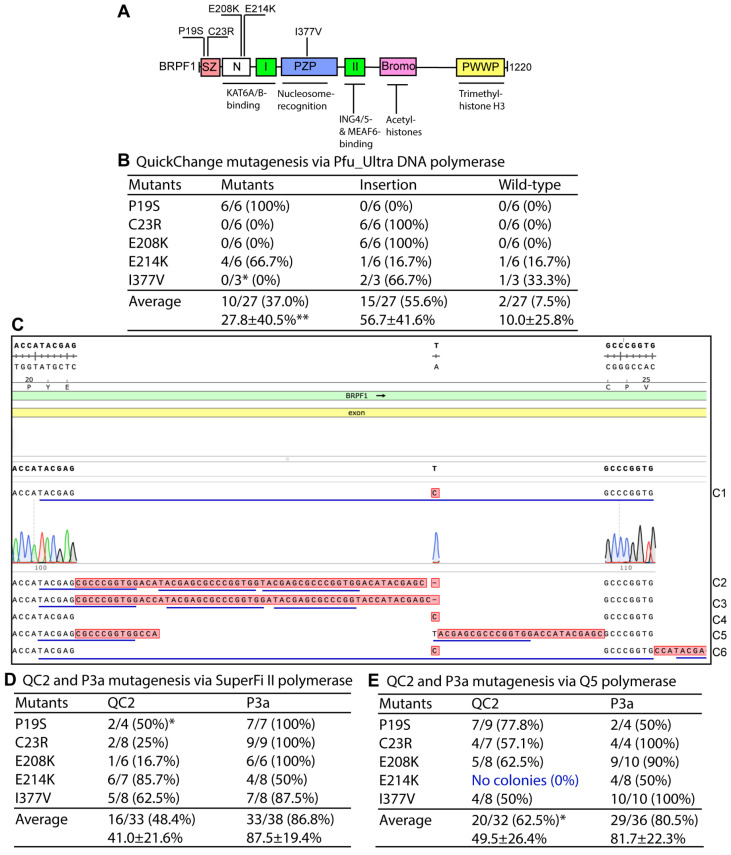
Comparison of different mutagenesis methods in engineering BRPF1 mutants. (**A**) Domain organization of human BRPF1. Five mutants to be engineered are indicated. Abbreviations: SZ, Sfp1-related zinc finger; N, the N-terminal region shared by BRPF1 and its related proteins; I and II, EPC1-homologous domains I and II, respectively; PZP, PHD-zinc-knuckle-PHD domain; Bromo, bromodomain; PWWP, Pro-Trp-Trp-Pro containing domain. (**B**) Efficiency of QuickChange mutagenesis when Pfu_Ultra DNA polymerase was used. An expression plasmid for human BRPF1 was used as the PCR template for QuickChange mutagenesis with Pfu_Ultra DNA polymerase. Notably, almost a half of the plasmids analyzed by sequencing possessed insertions at the primer sites. The single asterisk denotes that only three bacterial colonies were obtained under the same conditions, whereas the double asterisks refer to means and standard deviations calculated from the efficiency values for the five mutations. (**C**) Analysis of 6 plasmids sequenced for engineering the C23R mutant by the P3 method. All of them carried insertions at the primer sites. Moreover, the insertions share the 16-nucleotide repeat sequence TACGAGCGCCCGGTGG, derived from the primers used for mutagenesis. The repeats are underlined in blue. Inserts in clones #1, #3 and #6 are not (or not fully) shown in the figure: clone #1: GAG (Glu-26)→GA**CATACGAGCGCCCGGTGGA**G, where the insert is shown in bold and the 16-nucleotide repeat is underlined; clone #3: GAG (Glu-26)→GA**ATACGAGCGCCCGGTGGA**G; and clone #6: GAG (Glu-26)→CCATACGAGCGCCCGGTGGACCATACGAGCGCCCGGTGGACATACGAGCGCCCGGTGACCATACGAGCGCCCGGTGGACCATACGAGCGCCCGGTGGATACGAGCGCCCGGTG (6 repeats; 113 bp). (**D**) Efficiency of QC2 and P3 mutagenesis when SuperFi II DNA polymerase was used. An expression plasmid for human BRPF1 was used as the PCR template. As found with QuickChange with Pfu_Ultra DNA polymerase (panels (**B**,**C**)), most of the incorrect plasmids from QC2 mutagenesis possessed insertions at the primer sites. The asterisk denotes that only four bacterial colonies were obtained under the same conditions. The efficiency differences between the QC2 and P3a methods were statistically significant, with a two-tailed *p* value of 0.019 based on an unpaired Student’s T test. The success rate of 5/5 (i.e., 100%) from the QC2 method was also statistically different from that of 2/5 (40%) by the original QuickChange method (**B**), with a one-tailed *p* value of 0.003 according to a one-proportion Z test (the sample size was 5). (**E**) Efficiency of QC2 and P3 mutagenesis when Q5 DNA polymerase was used. As found with QuickChange with Pfu_Ultra DNA polymerase (panels (**B**,**C**)), many of the incorrect plasmids from QC2 mutagenesis possessed insertions at the primer sites. The asterisk denotes that no bacterial colonies were obtained for E214K under the same conditions; and this was not counted towards the average mutagenesis efficiency. The efficiency differences between the QC2 and P3a methods were not statistically significant, with a one-tailed *p* value of 0.07 based on an unpaired Student’s T test. The success rate of 4/5 (i.e., 80%) from the QC2 method was statistically different from that of 2/5 (40%) by the original QuickChange method (**B**), with a one-tailed *p* value of 0.034 according to a one-proportion Z test (the sample size was 5).

To leverage primer pairs with 3′-overhangs, we have recently developed the P3 site-directed mutagenesis method based on Pfu_Ultra [11,12]. The method reached an average efficiency of nearly 50% for many plasmid vectors [11,12]. For direct comparison with the QuickChange method, we also tested completely complementary primer pairs. For this, we decided to engineer mutants of a BRPF1 expression plasmid, which was previously used for developing the P3 site-directed mutagenesis method based on Pfu_Ultra [8,9]. The protein is composed of 1220 amino acid residues (Figure 1A), which is larger in size than most cellular proteins. We tested this protein because of our research interests in its role in epigenetic regulation and a neurodevelopmental disorder characteristic of intellectual disability and other symptoms [28,31,32,33,34,35,36,37]. As illustrated in Figure 1A, BRPF1 possesses multiple domains for epigenetic regulation, including the N-terminal part that interacts with lysine acetyltransferase 6A (KAT6A) and its paralog KAT6B [38,39,40]. In addition, BRPF1 possesses two EPC (Enhancer of Polycomb)-like motifs, the second of which interacts with ING4 (inhibitor of growth 4, or its paralog ING5) and MEAF6 (mammalian Esa1-associated factor 6) [28,39,40,41,42,43]. We sought to employ the QuickChange method to engineer five BRPF1 mutants (Figure 1A). As shown in Figure 1B, the efficiency varied dramatically, from 0% to 100%, dependent on the mutations. After analyzing 6 bacterial colonies per mutagenesis reaction, we could only obtain the P19S and E214K mutants, with an overall success rate of 2/5 (40%). By contrast, the success rate was ~100% for P3 or P3a mutagenesis [11,12,13]. These results support the advantage of partially complementary primer pairs employed in the P3 and P3a methods [11,12,13].

Intriguingly, instead of the presence of the wild-type plasmid, insertions at the primer sites were the main reason for the failure of the QuickChange method to engineer the C23R, E208K and I377V mutants (Figure 1B). The insertions were repeats of some sequences from the primers (Figure 1C). The insertions could be introduced either before the primers were annealed to the templates (via primer-primer false synthesis, such as dimerization) or after the strand synthesis reached the ends of the plasmid templates. The frequency of such insertions varied from mutation to mutation. For example, the C23R and E208K were much more prone to such insertions (Figure 1B,C). In stark contrast, the ideal mutagenesis efficiency of 100% was achieved for the P19S mutant (Figure 1B). Analysis of 6 plasmids sequenced for engineering the C23R mutant uncovered an interesting picture (Figure 1C). The insertions share the 16-nucleotide sequence TACGAGCGCCCGGTGG, which appears to be derived from the primers used for mutagenesis. Clone #6 possessed 6 copies of this 16-nucleotide sequence. The varied frequency of such insertions from mutation to mutation supports that their occurrence is dependent on the local sequence of the primer sites and the mutations to be engineered. This is in good agreement with the previous finding that certain BRPF3 mutations (such as R15W) were more difficult to engineer even with the P3 mutagenesis method [11].

We next asked whether replacement of Pfu_Ultra with SuperFi II DNA polymerase enhances the mutagenesis efficiency. The same five pairs of complementary primers (Figure 1A,B) were utilized. As shown in Figure 1D (middle), the efficiency increased substantially, reaching an average efficiency of 48.4%. All five mutants were easily obtained. For direct comparison, we also engineered the same five mutants with P3a mutagenesis via primer pairs with 3′-overhangs. The average efficiency was much higher, at 86.8% (Figure 1D, right), supporting the clear advantage of the P3 primer-designing strategy. It should be noted, however, that the QC2 method was much more efficient than the P3a method in engineering the E214K mutation (Figure 1D). It is possible that the local sequence around the mutation site dictates this unique outcome. Sequence analysis of the failed plasmids from the P3a method also identified frequent insertions at the primer sites. This rare case supports that the local sequence at the mutation site is an important factor affecting the mutagenesis efficiency.

We next asked whether replacement of Pfu_Ultra with Q5 DNA polymerase also enhances the mutagenesis efficiency. As shown in Figure 1E (middle), QC2 mutagenesis with Q5 DNA polymerase led to an average efficiency of 62.5% for four of the mutants (except for the E214K mutant, for which no colonies were obtained), supporting that replacement of Pfu_Ultra with Q5 DNA polymerase also enhances the mutagenesis efficiency of the QuickChange method. It should be noted that the QC2 method failed in engineering the E214K mutation. Overall, the average efficiency was lower than the P3a strategy with Q5 DNA polymerase (Figure 1E, right). Together with the findings with SuperFi II polymerase (Figure 1D), these results on Q5 polymerase (Figure 1E) indicate that although the efficiency was still lower than that from the P3a method, QC2 mutagenesis with SuperFi II or Q5 DNA polymerase is still reliable and outperforms the original QuickChange mutagenesis method, which is based on Pfu DNA polymerase and its derivatives (Figure 1B) [3,4,5,6].

### 3.2. QC2 Mutagenesis for Engineering Mutants of Three BRPF1-Related Epigenetic Regulators

**Figure 2:**
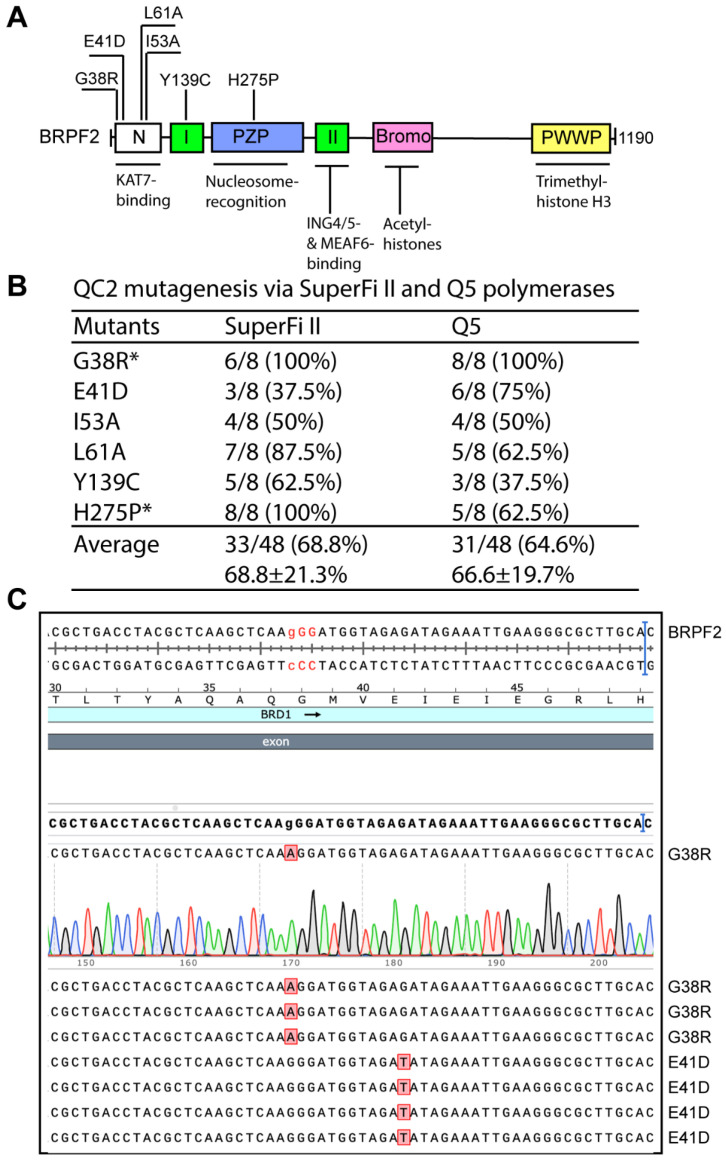
Engineering BRPF2 mutants via QC2 mutagenesis with SuperFi II and Q5 polymerases. (**A**) Domain organization of human BRPF2. Six mutants to be engineered are indicated. See Figure 1A for explanation of the abbreviations. (**B**) Efficiency of QC2 mutagenesis via SuperFi II DNA polymerase. An expression plasmid for human BRPF2 was used as the PCR template. As denoted by asterisks, the expression plasmid possesses two unwanted mutations (R38G and P275H), so the substitutions G38R and H275P were made to correct these two mutations. The efficiency differences between the two polymerases were not statistically significant, with a one-tailed *p* value of 0.28 based on an unpaired Student’s *T* test. (**C**) Analysis of 8 plasmids sequenced for engineering the G38R and E41D substitutions by (from QC2 mutagenesis via Q5 DNA polymerase.

To establish the general applicability of the QC2 mutagenesis method, we tested it with other expression plasmids. For this, we applied the method to expression vectors for BRPF1-related proteins. BRPF1 is highly homologous to BRPF2 and BRPF3, but KAT7 is a preferred partner of the latter two for acetylating nucleosomal histone H3 at lysine 14 [44,45,46]. By contrast, BRPF1 activates KAT6A and KAT6B [16]. Thus, BRPF1 is functionally different from BRPF2 and BRPF3. For BRPF2, we engineered six mutants via QC2 mutagenesis (Figure 2A). As shown in Figure 2B, SuperFi II and Q5 DNA polymerases yield average efficiencies of 68.8% and 64.6%, respectively. For some mutations, the efficiency reached 100%. However, the overall performance was still not comparable with P3a mutagenesis, which reaches ~100% [10].

**Figure 3:**
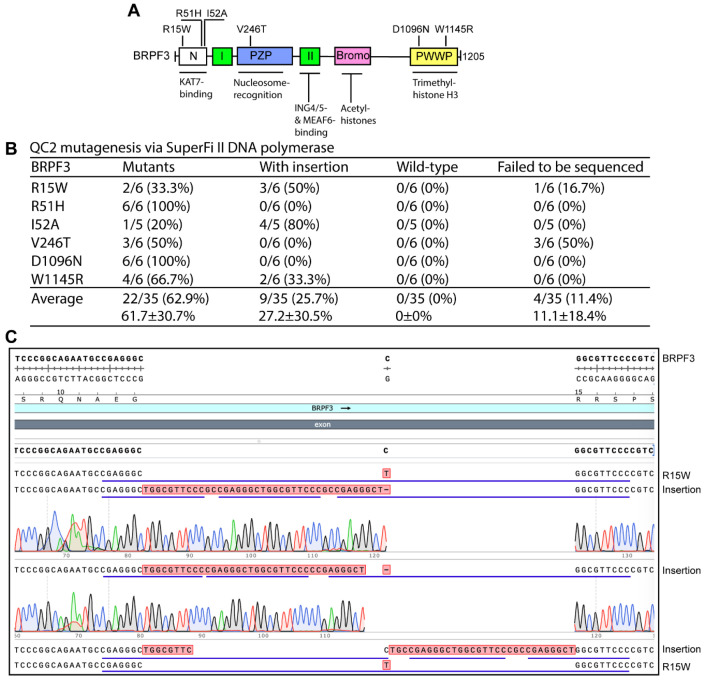
QC2 mutagenesis with SuperFi II polymerase for engineering BRPF3 mutants. (**A**) Domain organization of human BRPF3. Six mutants to be engineered are indicated. See Figure 1A for an explanation of the abbreviations. (**B**) Efficiency of QC2 mutagenesis via SuperFi II DNA polymerase. An expression plasmid for human BRPF3 was used as the PCR template. Notably, a quarter of the plasmids analyzed by sequencing possessed insertions at the primer sites. (**C**) Analysis of 6 plasmids sequenced for engineering the R15W mutant. Three of them possess insertions at the primer site. The insertions share a 14-nucleotide repeat (CGAGGTGGCGTTCC), underlined in blue, which appeared to be derived from the mutagenic primers.

For BRPF3, we also engineered six mutants via QC2 mutagenesis with SuperFi II DNA polymerase (Figure 3A), with an average efficiency of 62.9% (Figure 3B), which was much lower than that from P3a mutagenesis [10]. Many of the incorrect plasmids from QC2 mutagenesis possessed insertions at the primer sites (Figure 3B,C). For example, among 6 plasmids sequenced for the R15W mutant, three of them contained insertions that shared the 16-nucleotide repeat sequence CGAGGGCTGGCGTTCC, which appeared to be derived from the primers (Figure 3B). This is reminiscent of what was described above for BRPF1 (Figure 1).

**Figure 4:**
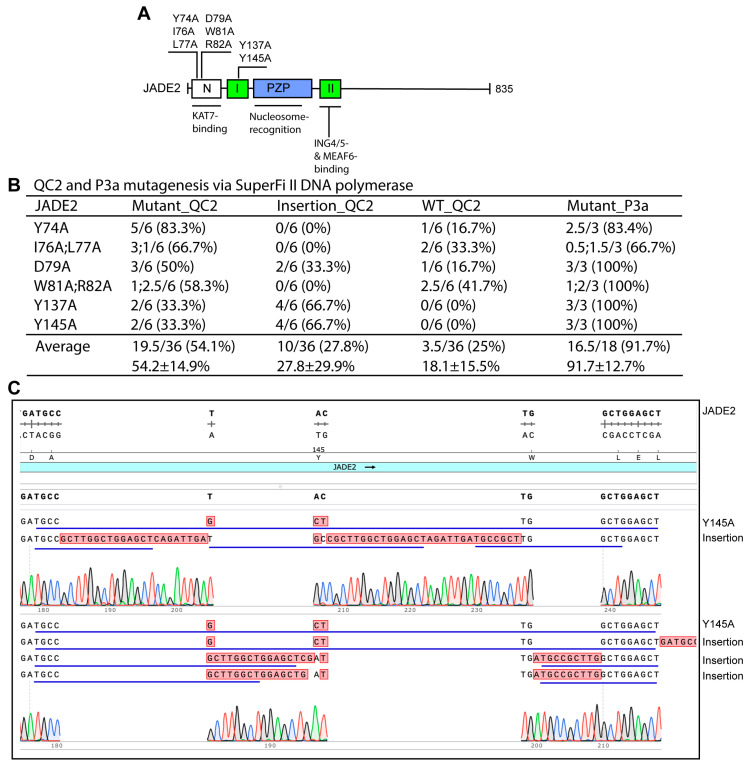
QC2 mutagenesis with SuperFi II polymerase to engineer JADE2 mutants. (**A**) Domain organization of human JADE2. Eight mutants to be engineered are indicated. See Figure 1A for an explanation of the abbreviations. (**B**) Efficiency of QC2 mutagenesis via SuperFi II DNA polymerase. An expression plasmid for human JADE2 was used as the PCR template. Notably, over a quarter of the plasmids analyzed by sequencing possessed insertions at the primer sites. The efficiency differences between the QC2 and P3a methods were statistically significant, with a one-tailed *p* value of 0.0016 based on an unpaired Student’s T test. (**C**) Analysis of 6 plasmids sequenced for engineering the Y145A mutant. Three of them possess insertions at the primer site. The insertions share a 18-nucleotide repeat (TGCCGCTTGGCTGGAGCT), underlined in blue, perhaps derived from the mutagenic primers.

We also utilized QC2 mutagenesis to engineer eight JADE2 mutants (Figure 4A). As shown in Figure 4A, JADE2 shares several domains with the BRPF family of epigenetic regulators, including the region mediating the interaction with KAT7 [47,48,49]. As shown in Figure 4B. the average efficiency of QC2 mutagenesis via SuperFi II DNA polymerase was 54.1%. Intriguingly, most of the incorrect plasmids from QC2 mutagenesis possessed insertions at the primer sites (Figure 4B,C). For example, three of the 6 plasmids sequenced for the Y145A mutant contained insertions that shared the 17-nucleotide repeat sequence GCCGCTTGGCTGGAGCT, which is likely derived from the primers (Figure 4B). This is similar to what was described above for BRPF1 (Figure 1) and its BRPF3 paralog (Figure 3).

### 3.3. QC2 Mutagenesis for Generating p300 Mutants

**Figure 5:**
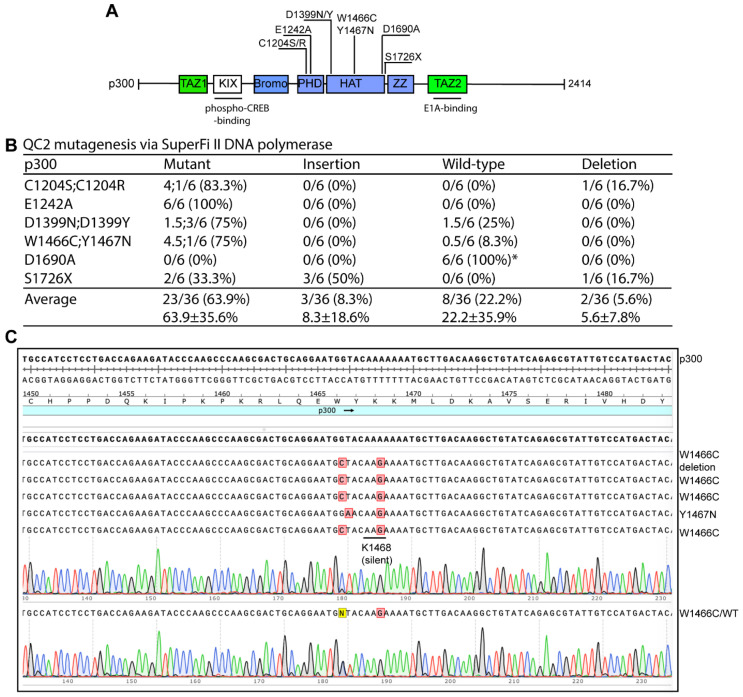
QC2 mutagenesis with SuperFi II polymerase for generating p300 mutants. (**A**) Domain organization of human p300. Nine mutants to be engineered are indicated. Abbreviations: TAZ1, transcriptional adapter zinc-binding domain 1; KIX, phospho-CREB-binding domain; bromo, bromodomain; PHD, plant homeodomain-linked zinc finger; HAT, histone acetyltransferase domain; ZZ, ZZ-type zinc finger; TAZ2, transcriptional adapter zinc-binding domain 2 [50,51,52,53]. (**B**) Efficiency of QC2 mutagenesis via SuperFi II DNA polymerase. An expression plasmid for human p300 was used as the PCR template. The term “C1204S;C1204R” denotes that these two mutants were engineered with a single pair of primers. (**C**) Analysis of 6 plasmids sequenced for engineering the W1666C and Y1467N mutants with a single pair of primers. A silent A→G mutation was introduced at the K1468 codon to break an A-stretch. Clone #1 possessed a deletion upstream from the mutation site, and clone #6 was a mixture with one allele for the wild-type and the other for W1466C.

In the superfamily of lysine acetyltransferases encoded by the human genome, p300 and CBP are the largest, each possessing ~2400 residues [50,51,52,53]. Their mammalian expression vectors reach 12.8 and 13.4 kb in size [13]. Because plasmid size is an important factor affecting PCR amplification and thus the efficiency of related mutagenesis methods, we tested a p300 expression plasmid with QC2 mutagenesis. For this, we sought to engineer nine p300 mutants, including D1399N, D1399Y, Y1414C, W1466C and Y1467N, which are five hotspot mutations in cancer [54,55]. The other mutations (such as C1204S/R, D1690A and the truncation mutant S1726X, where X refers to peptide termination from a stop codon) were selected to understand functions of different domains of p300, because C1204 and D1690 are key residues of two zinc fingers [56,57] and the truncation mutation removes the C-terminal one-third of the protein. As shown in Figure 5B, plasmids from 23 of the 36 colonies analyzed by Sanger sequencing carried the expected mutations, resulting in an average mutagenesis efficiency of 63.9%. Except for D1690A, the method was reliable for engineering the remaining eight p300 mutants (Figure 5A–C). Insertions at the primer sites were much less frequent although they were still an issue in engineering the S1726X mutant (50% containing insertions at the primer sites, Figure 5B). Overall, this efficiency was comparable to that from P3 mutagenesis [11] but still lower than what was obtained with the P3a method [13].

### 3.4. QC2 Mutagenesis of Two Expression Plasmids Possessing GC-Rich Sequences

**Figure 6:**
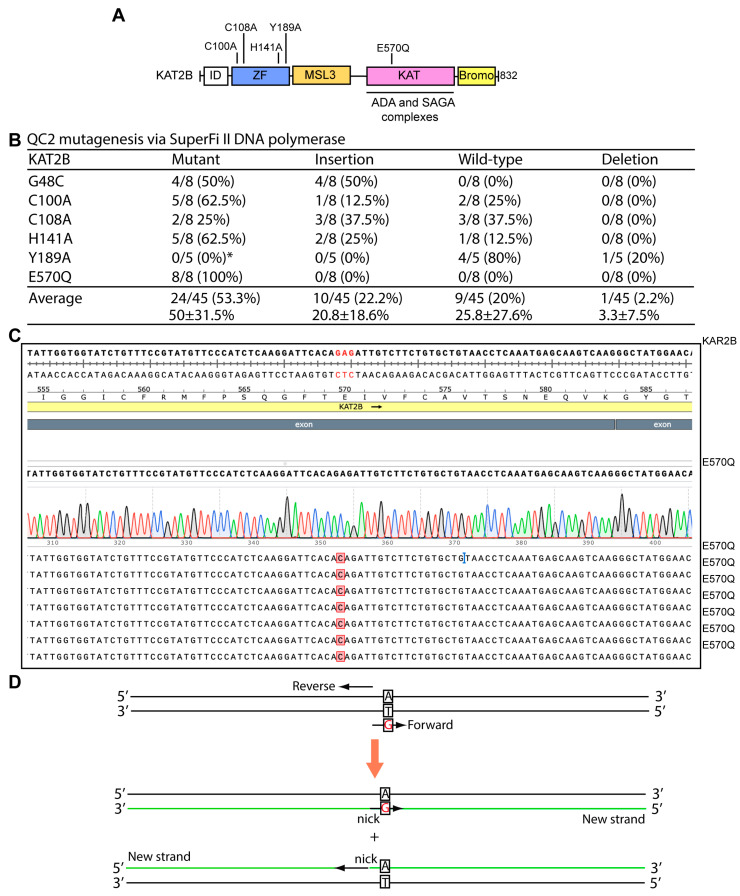
QC2 mutagenesis with SuperFi II polymerase in engineering KAT2B mutants. (**A**) Domain organization of human KAT2B. Five mutants to be engineered are indicated. Abbreviations: ID, intrinsically disordered domain; ZF, zinc finger; MSL3, MSL3-like domain; HAT, histone acetyltransferase domain; bromo, bromodomain [58,59,60,61,62,63]. (**B**) Efficiency of QC2 mutagenesis via SuperFi II DNA polymerase. An expression plasmid for human KAT2B was used as the PCR template as previously [21]. This plasmid contains two extremely GC-rich sequences in the CAG promoter and the coding sequence for the ID domain (panel (**A**)). These extremely GC-rich sequences render the plasmid incompatible with the PCR conditions described for P3a mutagenesis [21]. Thus, the P3b PCR conditions were used [21]. Notably, almost a quarter of the plasmids (i.e., 10 out of 45 analyzed) contained insertions at the primer sites. (**C**) Analysis of 8 plasmids sequenced for engineering the E570Q mutant. All yielded the correct mutation. (**D**) Primer-designing strategy in the Q5 mutagenesis method developed by New England Biolabs. Solid lines represent strands of a plasmid template used for mutagenesis, with the primers shown as arrows. The two primers are denoted as ‘Forward’, to indicate the forward primer containing a mutation that replaces the adenine nucleotide (A, black) on the template with G (red); and as ‘Reverse’, to refer to the reverse primer that possesses no mutations. Parental strands are susceptible to DpnI digestion due to their methylation at GATC sites, so after DpnI digestion and transformation, only the newly synthesized strands yield bacterial colonies. Only a half of the newly synthesized strands carry the mutation, so the maximal theoretical mutagenesis efficiency is 50%. At least one study reported that DpnI fails to digest an original parental strand if it is annealed to a newly synthesized strand and forms hemimethylated double strands [64], so the real efficiency should be lower than this maximal theoretical value.

Having established that QC2 mutagenesis is reliable for engineering mutations on the above five expression plasmids that possess regular GC-contents, we investigated how QC2 mutagenesis performs on plasmids possessing GC-rich sequences, which are known to pose problems to regular mutagenesis methods [11,21]. For this, we first tested the KAT2B expression plasmid that possesses two GC-rich regions known to impede P3 and P3a site-directed mutagenesis [11,21]. Complementary primer pairs were designed to engineer five mutants (Figure 6A), which have been tested for P3b mutagenesis [21]. As the two GC-rich regions known to impede P3a site-directed mutagenesis [8,12], the P3b PCR conditions were used with the five complementary primer pairs [21]. As shown in Figure 6B, the average efficiency was 53.3%, ranging from 0% (Y189A) to 100% (E570Q). About a quarter of the plasmids contained insertions at the primer sites (Figure 6B). Thus, except for Y189A, QC2 mutagenesis is still reliable, although the efficiency is much lower than P3b mutagenesis, supporting the superiority of primer pairs with 3′-protruding ends.

**Figure 7:**
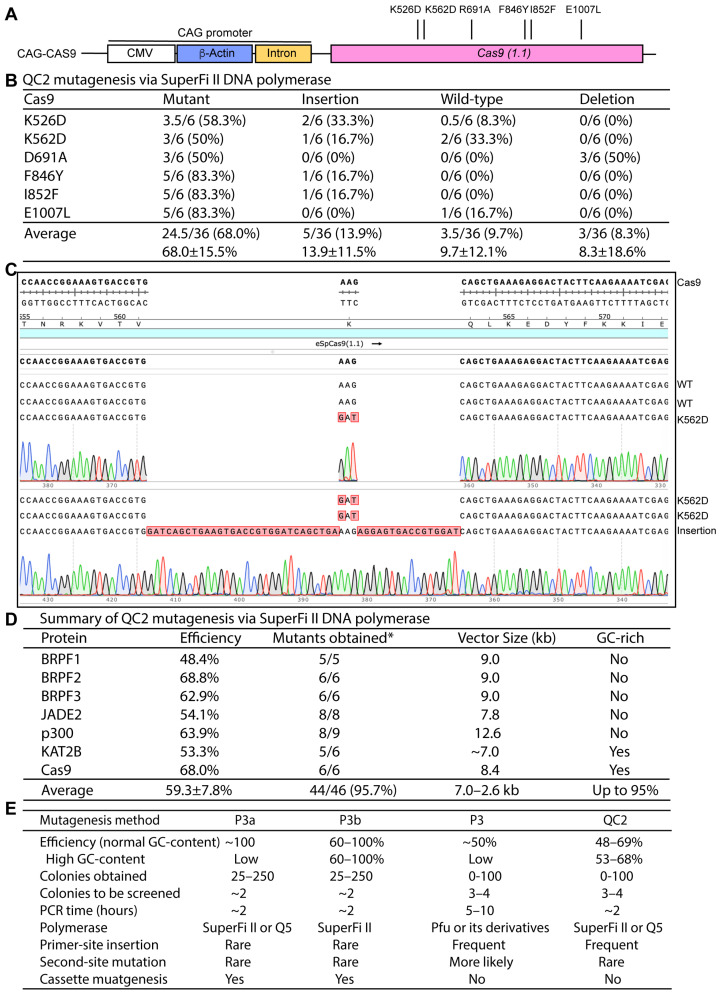
QC2 mutagenesis with SuperFi II polymerase to engineer Cas9 mutants. (**A**) Domain organization of Cas9 (1.1) [29,30]. Five mutants to be engineered are indicated. Upstream from the coding sequence is the CAG promoter, a synthetic promoter composed of a CMV promoter, a β-actin promoter and an intron [21]. The CAG promoter possesses GC-rich sequences that render the plasmid incompatible with the PCR conditions described for P3a mutagenesis, so the P3b PCR conditions were used instead [21]. (**B**) Efficiency of QC2 mutagenesis via SuperFi II DNA polymerase. An expression plasmid for human Cas9 (1.1) [29,30], as depicted in panel A, was used as the PCR template under the P3b conditions [21]. (**C**) Analysis of 6 plasmids sequenced for engineering the K562D mutant. Clone #6 possessed two insertions, which shared two repeats of a 27-nucleotide sequence (AGTGACCGTGGATCAGCTGAAAGAGGA), underlined in blue, identical to the primer site underlined in blue for clone #5. (**D**) Summary on the efficiency of QC2 mutagenesis in engineering 46 mutations on seven expression plasmids. The average efficiency reached 59.3%. The vector size ranged from 7.0 to 12.6 kb, with the GC-content reaching 95% for certain regions of the KAT2B and Cas9 expression vectors [21]. Overall, QC2 mutagenesis outperforms the classical QuickChange mutagenesis method (Figure 1B) and P3 site-directed mutagenesis [11,12]. Although the efficiency is still lower than that from the P3a and P3b methods [13,21], QC2 mutagenesis is still reliable. The asterisk refers to the success rate of 46 mutants that we attempted to engineer for seven proteins: All but two (D1690A of p300, Figure 5B; and Y189A of KAT2B, Figure 6B) were successfully engineered. (**E**) Comparison of the four different mutagenesis methods. The P3a and P3b methods are much more efficient and faster than P3 mutagenesis [11,13]. The risk of introducing unwanted mutation is also much lower with the P3a and P3b methods. The P3 and QC2 methods frequently introduce unwanted insertions and deletions at the primer sites. Compared to the P3 method, QC2 is faster and more efficient, but it is still less efficient than the P3a and P3b methods. Second-site mutations refer to misincorporation during strand extension, which is mainly determined by the fidelity of the DNA polymerase used and distinct from primer-site insertion. The colony number refers to colonies obtained with 1 μL of a mutagenesis reaction mixture transformed into 10 μL of DH5α competent cells.

We next investigated a Cas9 expression plasmid that also possesses GC-rich sequences. Six mutants were to be engineered (Figure 7A). Upstream from the coding sequence for Cas9 (1.1) is the CAG promoter, a synthetic gene-regulatory element composed of a CMV promoter, a β-actin promoter and an intron [29,30]. The CAG promoter possesses GC-rich sequences, which render the plasmid incompatible with the P3a PCR conditions [21]. As described above for KAT2B mutants, the P3b PCR conditions needed to be used [21]. As shown in Figure 7B, all six mutants were engineered successfully, with the mutagenesis efficiency ranging from 50% to 83.3%. The average efficiency reached 68% (Figure 7B). Among the incorrect plasmids, over a third possessed insertions at the primer sites (Figure 7B). Inspection of the insertions revealed their origin from the primers. For example, among the 6 plasmids sequenced for engineering the K562D mutant, clone #6 possessed two insertions composed of two repeats of a 27-nucleotide sequence (AGTGACCGTGGATCAGCTGAAAGAGGA), identical to the primer sites after the mutation was introduced (compared to clone #5, Figure 7C). These results reiterate that QC2 mutagenesis tends to introduce insertions at the primer sites with sequences derived from the primers. Together with the results obtained with the KAT2B expression plasmid, these findings demonstrate that QC2 mutagenesis is reliable for engineering mutants encoded by plasmid vectors with GC-rich sequences. Notably, the efficiency is still much lower than that from P3b mutagenesis with primer pairs containing 3′-overhangs [21], further supporting the advantage of such primers for site-directed mutagenesis.

## 4. Discussion

Even though site-directed mutagenesis was initially implemented over four decades ago [1,2], serious screening efforts for the correct mutants remain a common practice for many mutagenesis methods currently available, mainly due to two reasons: (1) overall efficiency is much lower than the ideal level of 100%, and (2) efficiency varies from mutation to mutation and from plasmid to plasmid. Reaching the efficiency at or close to 100% for all plasmids and mutations is thus an ideal goal for site-directed mutagenesis. Primer pairs with 3′-overhangs constitute an innovative site-directed mutagenesis strategy that involves utilizing primer pairs with 3′-overhangs [8,9,10]. Based on this strategy, we have recently developed P3, P3a and P3b mutagenesis methods, with the latter two allowing efficiency at or near 100% for almost all mutations that we have tested [11,13,21]. In addition to the primer design, another reason for the success is the use of SuperFi II and Q5 DNA polymerases, which are superior to Pfu in terms of fidelity, processivity and synthesis speed. This raises the interesting question whether these two polymerases also improve the performance of the classical QuickChange method [3,4,5,6]. Results presented herein support that this is indeed the case (Figure 7E). Except for a few cases, we could easily obtain 5–9 mutants for each of the seven tested plasmids in different sizes and with various GC-contents (Figure 7D). For the seven plasmids with sizes ranging from 7.0 to 12.6 kb and different GC-contents, the average efficiency varied from 48.4% to 68.8% (Figure 7D), which is much improved compared to the classical QuickChange method (Figure 1B). However, the efficiency is still much lower than that from P3a and P3b mutagenesis (Figure 7E) [10,12], further supporting the advantage using primer pairs with 3′-overhangs. Among the 46 mutations engineered, only two failed at the initial attempts (Figure 5B, Figure 6B and Figure 7D). This is much more reliable than the original QuickChange method (Figure 1B) [3,4,5,6]. By comparison, from the greater quantity of mutations that we have engineered with the P3a and P3b methods [10,12], none have failed, thereby reiterating the advantage of primer pairs with 3′-overhangs for site-directed mutagenesis. Nonetheless, the QC2 method still provides fast and reliable mutagenesis. Although we have only tested six epigenetic regulators and Cas9 due to their relevance to our own research program, this method should be applicable to many other genes and plasmids.

Moreover, compared to the classical QuickChange method [3,4,5,6], the PCR length for QC2 mutagenesis is much shorter (only about 2 h, Figure 7E). In this regard, QC2 mutagenesis is comparable to P3a and P3b mutagenesis [13,21]. For the classical QuickChange method with Pfu polymerase [2,3,4], the PCR length could reach over 10 h when a 13–14 kb plasmid is to be mutated. Thus, QC2 mutagenesis outperforms the classical QuickChange method in terms of efficiency and time saving. Moreover, the classical QuickChange method frequently failed to yield bacterial colonies. In comparison, this is much less of a problem with the QC2 method. Furthermore, due to the much higher fidelity of SuperFi II and Q5 DNA polymerases than Pfu and its derivatives, the QC2 method is expected to introduce secondary mutations (resulting from nucleotide misincorporation during DNA synthesis) at a much lower frequency (Figure 7E). Compared to complementary primer pairs used for QC2 mutagenesis, primer pairs with 3′-overhangs are longer, 30 vs. 20 nucleotides for a one-nucleotide mutation, so the primers for P3a and P3b mutagenesis are 50% more expensive than those for QC2 mutagenesis. For each QC2 mutagenesis mutation, 3–4 bacterial colonies need to be sequenced, but analysis of two bacterial colonies is typically sufficient with the P3a or P3b method (Figure 7E). If the plasmid preparation and sequencing costs are counted, the P3a and P3b methods remain advantageous over QC2 mutagenesis. Another advantage of the P3a and P3b methods is their ability to introduce cassette mutagenesis, which allows highly efficient deletion, insertion and replacement (Figure 7E) [13].

To our knowledge, no experimental efforts have been formally reported on the use of SuperFi II or Q5 DNA polymerase in the QuickChange method, which has been commercialized by Agilent Technologies. This method remains the most widely used in many laboratories around the world, owing in part to its maximal theoretical efficiency of 100%. New England Biolabs (Cat. E0554S, Ipswich, MA, USA) has marketed a site-directed mutagenesis kit based on Q5 DNA polymerase, but the primer-designing strategy is distinct from that described for the QuickChange method. The strategy used in the kit (Figure 6D) is not optimal as the maximal theoretical efficiency is only 50%. This is because only one primer of a pair used for mutagenesis contains the desired mutation and the other primer is wild-type. By comparison, the maximal theoretical efficiency is 100% for the QuickChange and P3 primer-designing strategies because both primers of a pair used for mutagenesis contain the given mutation. Thermo-Fisher Scientific has marketed a GeneArt site-directed mutagenesis system (Cat. A13282, Waltham, MA, USA), but its polymerase needs to be purchased separately. The vendor recommended AccuPrime™ Pfx DNA Polymerase (Cat. 12344024, Thermo Fisher Scientific, Waltham, MA, USA), whose fidelity is about 26-fold higher than that of Taq polymerase but still much lower than that of SuperFi II or Q5 DNA polymerase. The GeneArt mutagenesis procedure involves DNA methylation, PCR, subsequent fragment recombination and bacterial transformation, and is thus very different from (and also much more sophisticated than) P3 or QuickChange site-directed mutagenesis. In comparison, QC2 mutagenesis outperforms all these three methods. We have very recently noticed an application note from Thermo-Fisher Scientific on the usage of SuperFI II polymerase for site-directed mutagenesis. According to the note, only one pair of P3 primers was used with a *LacZ*-based assay, with no DNA sequencing performed, and no QuickChange primers were tested either.

**Figure 8:**
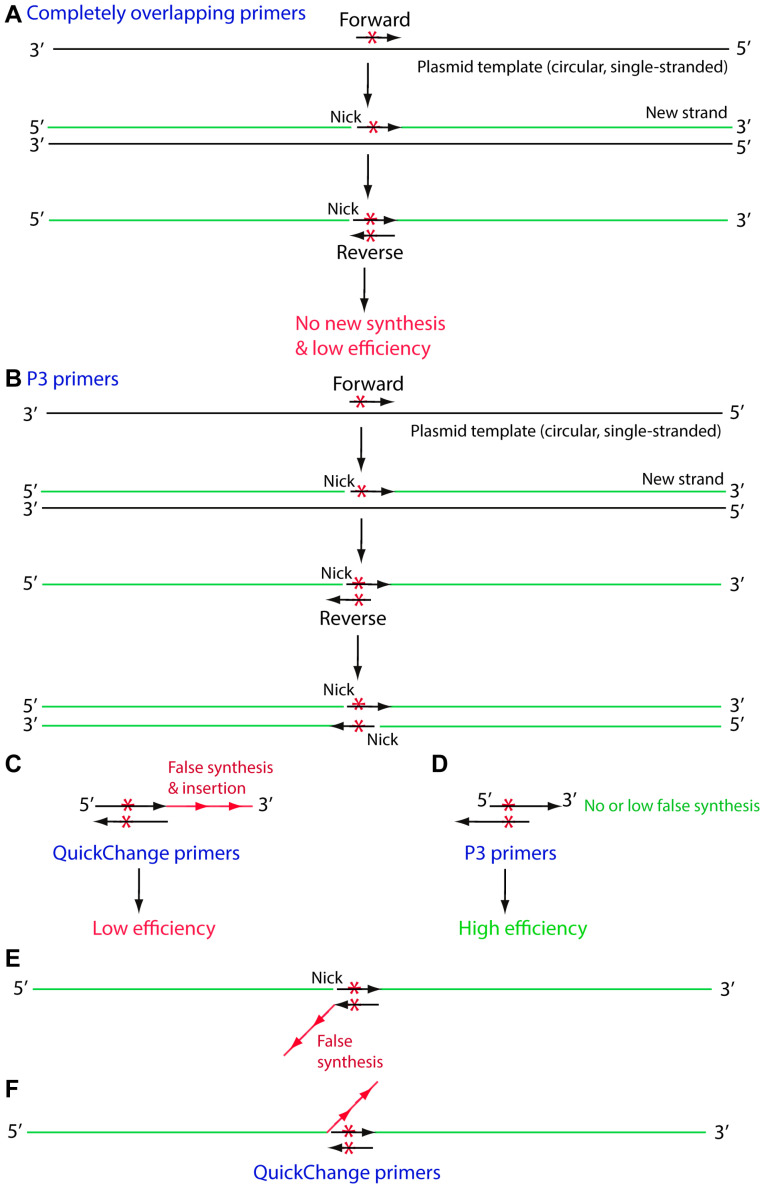
Comparison of completely and partially overlapping primer pairs used for mutagenesis. (**A**) Completely complementary primer pairs used in the QuickChange and QC2 methods fail to initiate DNA synthesis from newly synthesized strands replicated from the single-stranded parental plasmid templates generated from heat denaturation. (**B**) Partially complementary primer pairs with 3′-overhangs, employed in the P3, P3a and P3b methods, are capable of initiating DNA synthesis from newly synthesized strands replicated from the denatured parental plasmid templates. This has been considered as the major reason that these methods are superior to the QuickChange strategy in terms of mutagenesis efficiency [5,6,8,9,10]. But no solid experimental proofs have been reported on this. Instead, the results from this study identify an alternative mechanism: insertion of primer-derived short repeats at the primer sites dramatically decreases the efficiency of mutagenesis methods based on completely complementary primer pairs. (**C**) Completely complementary primer pairs used in the QuickChange and QC2 methods may initiate false synthesis, introduce insertions at primer sites and reduce mutagenesis efficiency. For example, primer dimers or oligomers are formed in such a manner. (**D**) Partially complementary primer pairs, with 3′-overhangs, may not initiate erroneous synthesis, thereby leading to higher mutagenesis efficiency than the QuickChange and QC2 methods. (**E**) False synthesis occurs at the 3′-end of a primer annealed to a newly synthesized strand. The incorrect primer then initiates strand extension and thereby introduces the insertion. (**F**) Erroneous synthesis occurs when the new strand reaches the 5′-end of an annealed primer. After bacterial transformation, the insertion is kept during nick repair.

For the P3, P3a and P3b mutagenesis methods, an accepted notion is that primer pairs with 3′-overhangs enhance mutagenesis efficiency by allowing the use of newly synthesized strands as templates for subsequent PCR amplification (Figure 8A,B) [8,9,10,13]. However, there is no solid experimental evidence supporting this notion. By contrast, sequence analysis of the incorrect plasmids from QC2 mutagenesis revealed frequent insertions of oligonucleotide repeats at the primer sites, with certain primer sites much more prone to such insertions, perhaps due to their unique sequences (Figure 1B,C, Figure 3B,C, Figure 4B,C and Figure 7B,C). Such insertions have also been observed in the previous studies on the P3 method using Pfu polymerases and their derivatives, but these insertions were rare with the P3a and P3b methods, in which SuperFi II or Q5 DNA polymerase was employed [11,13,21], indicating that the choice of DNA polymerase is one major factor affecting the occurrence of such insertions. The high frequency of such insertions with the QC2 method indicates that primer design is another factor that affects the occurrence of such insertions. A third factor is the local sequence of the mutation sites, as supported by the varied frequency at different mutation sites even for the same genes and mutagenesis methods (Figure 1B,C, Figure 3B,C, Figure 4B,C and Figure 7B,C). Together, the new findings that such insertions also occurred with the QC2 method and decreased its efficiency indicate that primer pairs with 3′-overhangs in the P3a and P3b methods enhance mutagenesis efficiency largely because of the low likelihood to introduce insertions at the primer sites (Figure 8C,D).

This newly identified mechanism is completely different from the accepted notion that primer pairs with 3′-overhangs enhance mutagenesis efficiency by allowing the use of newly synthesized strands as templates for subsequent PCR amplification (Figure 8A,B) [5,6,8,9,10]. This new mechanism also explained nicely the rare case where the QC2 method outperformed the P3a method (E214K, Figure 1D). By contrast, this rare case would be challenging and difficult to comprehend when only considering the original model that primer pairs with 3′-overhangs enhance mutagenesis efficiency by allowing the use of newly synthesized strands as templates for subsequent PCR amplification (Figure 8B). The insertions share sequences with primers (e.g., Figure 1C, Figure 3C, Figure 4C and Figure 7C) and appear to be derived from them, suggesting that repeated synthesis resulting from primer sliding is one potential mechanism whereby the insertions are introduced. Hypothetically, there are three other possibilities, as illustrated in Figure 8C,E,F. At this moment, it is unclear which one(s) really occurs. Therefore, the current study also uncovers an unexpected mechanism underlying the high efficiency of P3a and P3b mutagenesis, which sheds new light on how to improve the QC2 method further. Moreover, this raises an interesting question whether it is possible to shorten the P3a and P3b primers (e.g., to 24 or 25 nucleotides) and still maintain the high mutagenesis efficiency.

In summary, we have developed QC2 mutagenesis by replacing Pfu polymerase in the original QuickChange method with two superior DNA polymerases and systematically evaluated the QC2 mutagenesis method with 46 mutations across seven different plasmids of varying sizes and GC-contents (Figure 7D). Compared to the original QuickChange method (Figure 1A–C), this QC2 method is more efficient and takes much less time (Figure 1D,E). The average efficiency for engineering 46 mutations on seven expression plasmids was 59.3% (Figure 7D). With these 46 mutations, we obtained 44 of them at the initial attempts (Figure 7D), so the method is much more reliable than the P3 method (Figure 1A–C). However, the average efficiency of 59.3% (Figure 7D) is still much lower than that from the P3a or P3b method (Figure 1D,E and Figure 4B) [13,21]. Notably, the primers used in the QC2 method are 50% shorter than those for P3a and P3b mutagenesis and thus cost less. However, due to higher efficiency, plasmids from only two bacterial colonies need to be sequenced per mutagenesis reaction from the P3a or P3b method [13,21], whereas 3–4 bacterial colonies should be analyzed per mutagenesis reaction from the QC2 method (Figure 7E). Thus, the QC2 method still costs more than the P3a or P3b method. Furthermore, sequences of the incorrect plasmids from the QC2 method revealed frequent insertions at the primer sites (Figure 1C, Figure 3C, Figure 4C and Figure 7C), thereby uncovering a novel mechanism whereby primer pairs with 3′-overhangs promote high-efficiency mutagenesis at least in part by reducing the likelihood of introducing insertions at the primer sites (Figure 8C,D). It will be interesting to investigate whether it is feasible to eliminate such insertions and improve the QC2 mutagenesis efficiency further.

## 5. Conclusions

Herein, we have demonstrated that the use of SuperFi II and Q5 DNA polymerases improves both the efficiency and reliability of QuickChange site-directed mutagenesis (Figure 7E). We have also found that incorrect plasmids from the improved QuickChange method frequently possess insertions at primer sites. Such insertions were hardly found with the P3a and P3b method, so primer pairs with 3′-overhangs enhance mutagenesis efficiency by minimizing insertions at primer sites. This is a novel mechanism by which primer pairs with 3′-overhangs enhance mutagenesis efficiency (Figure 8C,D), which, hopefully, will help us leverage this newly acquired knowledge for optimizing site-directed mutagenesis further in the near future. Therefore, the current study not only improves QuickChange mutagenesis and supports the superiority of the P3a and P3b methods [13,21], but also uncovers a new molecular mechanism by which the efficiency of PCR-based mutagenesis with partially or completely overlapping primer pairs is governed.

## Data Availability

Data and research materials are available from the corresponding author upon reasonable request.

## Supplementary Materials

The following supporting information can be downloaded at: https://www.mdpi.com/article/10.3390/cells15020138/s1, Table S1: Primers to engineer mutants of six epigenetic regulators and Cas9.
