# Supplementary material for: Efficiency and Fidelity of Site-Directed Mutagenesis with Complementary Primer Pairs
Source: Cells. 2026 Jan 13;15(2):138. doi: 10.3390/cells15020138 (PMC12839903; doi:10.3390/cells15020138)
Supplement: Supplementary file 1 [file cells-15-00138-s001.zip › cells-4057342-Supplementary file.pdf]

Table S1. Primers to engineer mutants of six epigenetic regulators and Cas9.

Mutation	Forward primer	Reverse Primer
	BRPF1 (-F/R)	
P19S	GCGACTAAGtCACCATACGAGTGCCCGGTG	CGTATGGTGaCTTAGTCGCCCGCAAGTTGT
C23R	CCATACGAGcGCCCGGTGGAGACCTGCCGAAA	CCACCGGGCgCTCGTATGGTGGCTTAGTCGCC
E214K	GGACGAGGAAGTAaAGTATGACAT	ATGTCATACTtTACTTCCTCGTCC
I377V	CATTGAGCACgTCCCACCAGC	GCTGGTGGGAcGTGCTCAATG
	BRPF1 (-F1/R1)	
P19S	GGCGACTAAGtCACCATACGA	TCGTATGGTGaCTTAGTCGCC
C23R	ACCATACGAGcGCCCGGTGGA	TCCACCGGGCgCTCGTATGGT
E208K	GTCTGCAGAGaAGCTGGACGA	TCGTCCAGCTtCTCTGCAGAC
E214K	GAGGAAGTAaAGTATGACATGGACGAGGAG	TGTCATACTtTACTTCCTCGTCCAGCTCCT
	BRPF1 (-F2/R2)	
E208K	TCTGCAGAGaAGCTGGACGAGGAAGTAGAGTATGACATGG ACGAG	CGTCCAGCTtCTCTGCAGACTTCTCGATGTACCGGTAATAG GAAG
I377V	ATTGAGCACgTCCCACCAGCTCGCTGGAAGCTCACCTGCTA CATT	CTGGTGGGAcGTGCTCAATGCTGTCAATAGGCTCTAGGAA GACCG
	BRPF1 (-F3/R3)	
E208K	TCTGCAGAGaAGCTGGACGAGGAAGTAGAG	CGTCCAGCTtCTCTGCAGACTTCTCGATGT
I377V	ATTGAGCACgTCCCACCAGCTCGCTGGAAG	GCTGGTGGGAcGTGCTCAATGCTGTCAATA
	BRPF2 (-F1/R1)	
G38R	CTCAAGCTCAAaGGATGGTAGAG	CTCTACCATCCtTTGAGCTTGAG
E41D	GGATGGTAGAtATAGAAATTG	CAATTTCTATaTCTACCATCC
I53A	CAGGATCAGTgcTTTTGATCCCCT	AGGGGATCAAAAgcACTGATCCTG
L61A	TGGAGATCATAgcGGAAGATGAC	GTCATCTTCCgcTATGATCTCCA
Y139C	CCTCCTGTGTgCTACAAGTTC	GAACTTGTAGcACACAGGAGG
H275P	TGTGCTGTGCCCCAACAAGGGTG	CACCCTTGTTGGGGCACAGCACA
	BRPF3 (-F1/R1)	
R15W	TGCCGAGGGCtGGCGTTCCC	GGGAACGCCaGCCCTCGGCA
R51H	GCCTGCATCaTATCAGCATC	GATGCTGATAtGATGCAGGC
I52A	GCCTGCATCGTgcCAGCATCTATG	CATAGATGCTGgcACGATGCAGGC
V246T	GTGCTATGGCaTCCCATACATC	GATGTATGGGAtGCCATAGCAC
D1096N	CTTGATCATCaATCCCAAGATG	CATCTTGGGATtGATGATCAAG
W1145R	CAAGCGCACCaGGCAGTGGCT (W1145R-F3)	AGCCACTGCCtGGTGCGCTTG (W1145R-R3)
	JADE2 (-F/R)	
Y74A	GCCCGGATGACgctCTACATCCTGGCAGACCCATGGC	CTGCCAGGATagcGTAGTCATCCGGGCTGAGCTGGT
I76A	TGACTACTACgctCTGGCAGACCCATGGCGACAGGA	ATGGGTCTGCagcGATGTAGTAGTCATCCGGGCTGA
W81A	TGGCAGACCCAgctCGACAGGAATGGGAGAAAGGTG	CCATTCCTGTgcCCATGGGTCTGCCAGGATGTAGTA (R82A-R)
D79A	ACATCCTGGCAgctCCATGGCGACAGGAATGGGA	TGTCGCCATGGagcTGCCAGGATGTAGTAGTCAT

Y137A	GGGCAGCCGCgcTGACTTGGACGAGATTGATG	GTCCAAGTCAgcGCGGCTGCCCCCTGGCCAAT
Y145A	AGATTGATGCCgctTGGCTGGAGCTCATCAACTC	AGCTCCAGCCAagcGGCATCAATCTCGTCCAAGT
	**JADE2 (-F1/R1)**	
Y74A	GCCCGGATGACgctCTACATCCTGGC	GCCAGGATGTAGagcGTCATCCGGGC (Y75A-R1)
I76A	TGACTACTACgctCTGGCAGACCCAT	ATGGGTCTGCagcGATGTAGTAGTCA (L77A-R1)
D79A	ACATCCTGGCAgctCCATGGCGACAG	CTGTCGCCATGGagcTGCCAGGATGT
W81A	TGGCAGACCCAgctCGACAGGAATGG	CCATTCCTGTgcCCATGGGTCTGCCA (R82A-R)
Y137A	GGGGCAGCCGCgcTGACTTGGACGA	TCGTCCAAGTCAgcGCGGCTGCCCC
Y145A	AGATTGATGCCgctTGGCTGGAGCTC	GAGCTCCAGCCAagcGGCATCAATCTC
	**p300 (-F1/R1)**	
C1204S/R	TGTGAGAAGTcTTTCAATGAGAT	ATCATTGAAACgCTTCTCACAGA
E1242K/A	CACTGGATCCTaAACTGTTTGTT	AACAAACAGTgCAGGATCCAGTG
D1399Y/N	ATCTTACCTCtATAGTGTTCATT	AATGAACACTATtGAGGTAAGAT
W1466C/Y1467N	GCAGGAATGcTACAAgAAAATGCTTG (W1466C-F1)	CAAGCATTTTcTTGTtCCATTCCTGC (Y1467N-R1)
D1690A	TGAGGATTATGcCTTGTGTATCA	TGATACACAAGgCATAATCCTCA
S1726X	CAGCCACCCAGtAGCCCAGGCGA	TCGCCTGGGCTaCTGGGTGGCTG
	**KAT2B (-F1/R1)**	
G48C	TGCCGCCGGGtGCTCGGGCGCC	GGCGCCCGAGCaCCCGGCGGCA
C100A	GTGTACTCCGCCgctAAGGCCGAGGA	TCCTCGGCCTTagcGGCGGAGTACAC
C108A	AGTCTTGTAAAgcTAATGGCTGGAA	TTCCAGCCATTAgcTTTACAAGACT
H141A	CGGAGTTGTAGCgcTGCCCTAGCTGC	GCAGCTAGGGCAgcGCTACAACTCCG
Y189A	CAAGTTTATTTCgcTCTATTTAAGC	GCTTAAATAGAgcGAAATAAACTTG
E570Q	AGGATTCACAcAGATTGTCTTC	GAAGACAATCTgTGTGAATCCT
	**Cas9 (-F1/R1)**	
K526D	AACGAGCTGACCgatGTGAAATACGTG	CACGTATTTCACatcGGTCAGCTCGTT
K562D	AAAGTGACCGTGgatCAGCTGAAAGAGG	CCTCTTTCAGCTGatcCACGGTCACTTT
R691A	GGCTTCGCCAACgccAACTTCATGCAGC	GCTGCATGAAGTTggcGTTGGCGAAGCC
F846Y	GCCTCAGAGCTaTCTGGCCGACG	CGTCGGCCAGAtAGCTCTGAGGC
I852F	CGACGACTCCtTCGACAACAAGG	CCTTGTTGTCGAaGGAGTCGTCG
E1007L	GCGCTGGAAAGCctGTTCGTGTACGG	CCGTACACGAACagGCTTTCCAGCGC

Note: Mutated nucleotides are shown in lowercase. Because they were designed at the initial stage of the project to develop P3 site-directed mutagenesis, E208K-F2/R2 and I377V-F2/R2 are much longer than typical P3 primers that we used, We also tested E208K-F3/R3 and I377V-F3/R3, which are P3 primers of typical length.
